# Clinical management of a pregnant woman with Filamin C cardiomyopathy

**DOI:** 10.2459/JCM.0000000000001294

**Published:** 2022-01-10

**Authors:** Riccardo Bariani, Giulia Brunetti, Alberto Cipriani, Ilaria Rigato, Rudy Celeghin, Monica De Gaspari, Kalliopi Pilichou, Barbara Bauce

**Affiliations:** Department of Cardiac, Thoracic, Vascular Sciences and Public Health, University of Padua, Padua, Italy

Detection of ventricular arrhythmias and left ventricular (LV) dimensional and kinetic abnormalities in a young patients raises the issue of a correct diagnostic workup. This situation becomes more complicated if we are dealing with a pregnant woman, considering that safety for the fetus of some fundamental diagnostic tools, such as cardiac magnetic resonance (CMR) with contrast agent, has not been established yet. Here we describe the diagnostic process and the clinical management of this patient.

A 34-year-old primiparous woman at sixth week of pregnancy, previously asymptomatic, complained of palpitations at rest. Family history reported a diagnosis of dilated cardiomyopathy (DCM) in the mother with Implantable cardioverter-defibrillator (ICD) implant for recurrent episodes of sustained ventricular tachycardia. The mother died later because of a neoplastic disease. Despite the family history of DCM, the patient did not have a previous cardiac evaluation. Moreover, the family was of a small size and no other family members were available for clinical study or were reported to be affected by an arrhythmic cardiac disease.

At first evaluation, 12-lead ECG showed sinus rhythm, low QRS voltages in limb leads, repolarization abnormalities in precordial leads and presence of premature ventricular beats (PVBs) (Fig. 1a). Two-dimensional Doppler echocardiography revealed a LV dilatation (LV end-diastolic volume = 90 ml/m^2^) with ejection fraction at lower normal limits (50%) and presence of kinetic alterations localized in the posterior wall. The right ventricle (RV) was mildly dilated with normal systolic function.

**Fig. 1:**
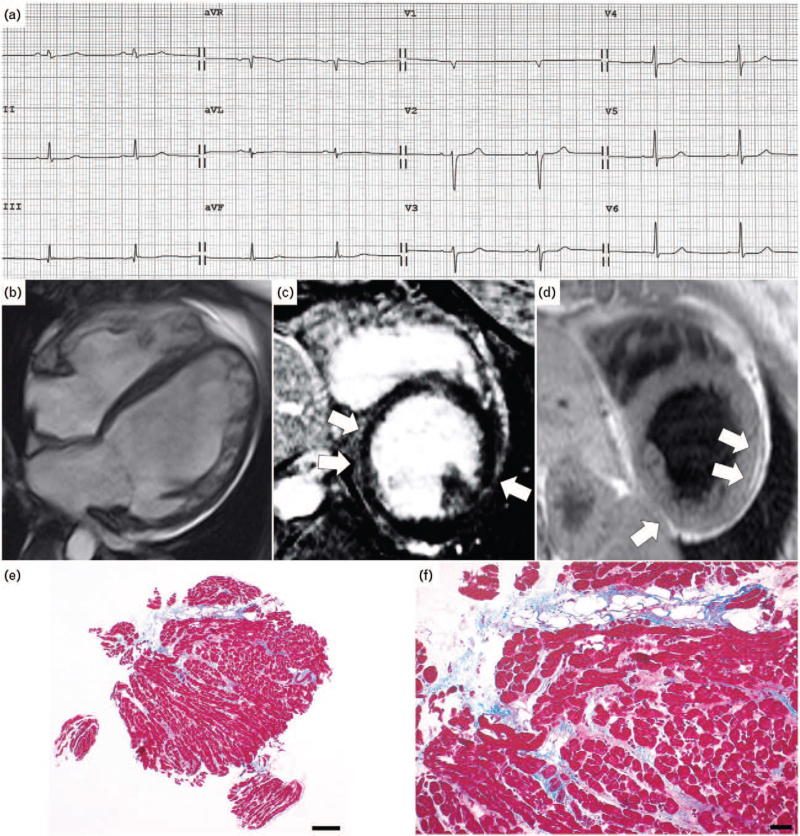
(a) ECG at admission showing sinus rhythm, low QRS voltages in limb leads, repolarization abnormalities in precordial leads and presence of premature ventricular beats; (b–d) contrast-enhanced CMR, (b) cine image with severe dilatation of LV and mild LV systolic dysfunction, mild dilatation of RV with preserved EF; (c) proton density weighting sequence showing fat infiltration of LV, involving mid segments of inferior and lateral walls (arrows); (d) postcontrast sequences showing a nonischemic subepicardial LGE in inferior and mid lateral walls (arrows); (e and f) endomyocardial biopsy with focal fibrofatty replacement of the myocardium [Heidenhain trichrome stain, scale bar 100 μm for (e) and 50 μm for (f)]. CMR, cardiac magnetic resonance; EF, ejection fraction; LV, left ventricular; RV, right ventricle.

A 24-h Holter monitoring showed sinus rhythm, numerous polymorphic PVBs with prevalent morphology of right bundle branch block Right bundle branch block (RBBB) with superior axis deviation. Routine blood biomarkers, including Troponin I and BNP, were within limits and a mild hypokalemia was the only altered parameter. She was treated with beta-blocker therapy (metoprolol 50 mg twice daily) and hypokalemia correction and was followed up in cooperation with the Obstetrics Department.

At the 11th week of pregnancy, she was admitted to our Cardiologic department because of detection at 24-h Holter monitoring of numerous polymorphic PVBs and a few runs of non-sustained ventricular tachycardia (NSVT) [max 18 beats, heart rate (HR) 148 bpm] with RBBB and superior axis deviation (Fig. 2). Pharmacological therapy was titrated (metoprolol 100 + 50 mg/day) with decreasing PVB frequency and complexity. At 2D echocardiogram, LV dilatation resulted to be increased (LV end-diastolic volume = 100 ml/m^2^) while systolic function was mildly reduced (ejection fraction = 46%). RV dimension and function were unchanged. During the following weeks, the patient was asymptomatic and instrumental findings remained stable with recording of isolated PVBs.

**Fig. 2:**
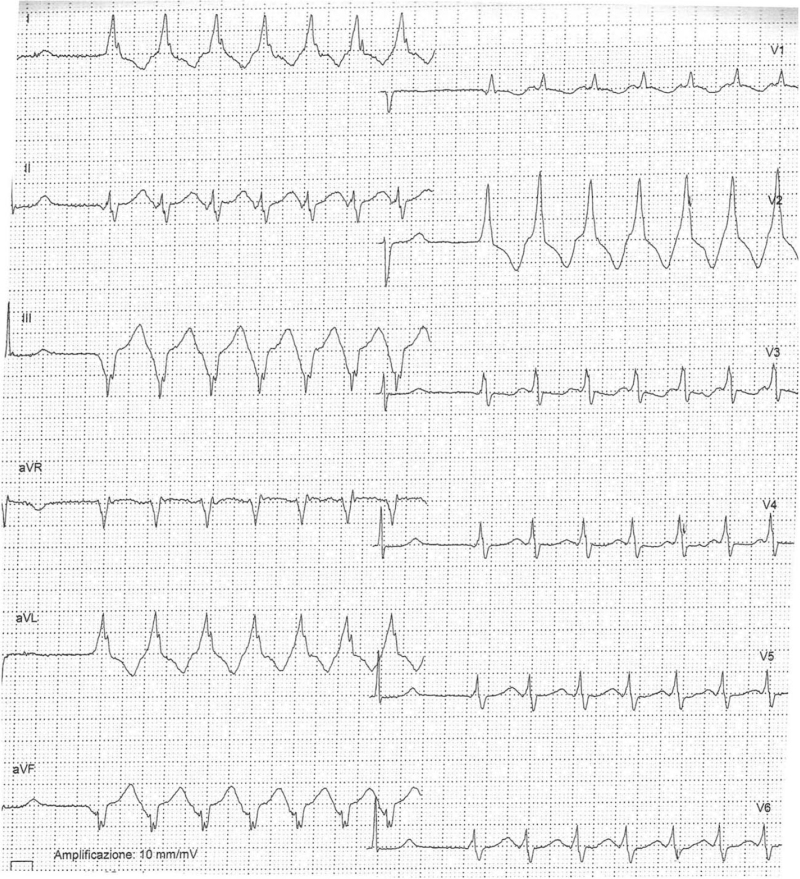
Strip from Holter ECG monitoring showing a run of nonsustained ventricular tachycardia with RBBB morphology. RBBB, right bundle branch block.

At the 38th week of pregnancy for obstetric evidence of fetal growth delay she underwent a programmed delivery by caesarean section. After a short monitoring in our intensive unit, lactation was suppressed (Cabergoline 0.25 mg twice daily for 2 days) and an ACE inhibitor (Ramipril 2.5 mg) was added to beta-blocker therapy. Finally, a complete diagnostic cardiological workup was carried out. CMR showed a moderate LV dilation with mild systolic dysfunction and segmental abnormalities on inferior and lateral walls. The RV was mildly dilated with normal systolic function. Moreover, a LV epicardial fibrofatty substitution involving inferior and mid lateral walls was present (Fig. 1b–d). Cardiac catheterization demonstrated normal coronary arteries. Right ventricular endomyocardial biopsy showed focal fibrofatty replacement of the myocardium, in the absence of inflammatory infiltrates (Fig. 1e and f); PCR was negative for cardiotropic viruses. The patient was finally diagnosed with AC with left dominant phenotype and discharged from the hospital with unchanged medications. In the following week, she complained of palpitations with a presyncopal episode at rest and an ICD was implanted. Six months later genetic test revealed the presence of a pathogenic mutation of Filamin C (c.7037dup, p.Leu2347Profs∗9).

Identification of an inherited arrhythmic disease characterized by a dilated LV with impaired systolic function leads to the need to make a differential diagnosis mainly between DCM and AC with left dominant phenotype.^1–3^ It is noteworthy that also myocarditis may show a phenotypic overlap with both forms of cardiomyopathies, even if family history of cardiomyopathy is less in keeping with this diagnosis.^4^

In our patient, arrhythmic symptoms occurred in the first weeks of pregnancy and a significant degree of electrical instability characterized the clinical onset of the disease.

Twelve-lead ECG findings with precordial repolarization abnormalities and low QRS voltages in limb leads were more consistent with an AC with left dominant phenotype or a myocarditis form.^5,6^ Echocardiographic evaluation documented the presence of moderate LV dilation with a mildly reduced systolic function with kinetic abnormalities and a mild dilatation of RV with normal function. These findings could be in keeping with both DCM, AC with left dominant phenotype and myocarditis diagnosis, even if the presence of regional kinetic abnormalities is less common in DCM.^1,5^ Moreover, in our patient, the pregnancy condition had important implications in the diagnostic assessment as CMR with contrast agent should be usually avoided during pregnancy for possible fetal effects.^6^ In our subject, the detection of subepicardial fibrofatty substitution in inferior and mid lateral walls was in keeping with both the diagnosis of AC and myocarditis. Augusto *et al.* found a specific CMR LGE pattern, characterized by circumferential subepicardial involvement (the so-called ‘ring pattern’) in 57% of the FLNC population. Moreover, in 87% of *DSP/FLNC* mutation carriers, LGE was localized on subepicardial basal lateral segments.^7^ According to these findings, in our patient, we found a nonischemic subepicardial LGE distribution in inferior and mid lateral walls (Fig. 1). Finally, endomyocardial biopsy documented the presence of focal fibrofatty tissue and genetic analysis revealed a pathogenic mutation of the *Filamin C* gene was identified.

A peripartum cardiomyopathy (PPC) was excluded because of the onset of symptoms and detection of ventricular abnormalities in the first trimester of pregnancy. The current definition of PPC includes the development of heart failure toward the last trimester of pregnancy and the first 6 months postpartum, in the absence of other identifiable causes, in the presence of an EF LV less than 45%.^8^ Furthermore, in this case, the clinical presentation was characterized by symptomatic ventricular arrhythmias rather than heart failure.

Family screening plays an essential role in the diagnosis of cardiomyopathy. Also in our case, family history of cardiomyopathy was helpful in differential diagnosis with an acquired cardiac disease. It is noteworthy that our patient, despite a family history of DCM, did not undergo previous cardiac evaluation that could have allowed an early diagnosis. Even if she had a stable hemodynamic condition, the abrupt onset of arrhythmic symptoms with detection of frequent NSVT episodes induced us to rule out the presence of a chronic myocarditis through an endomyocardial biopsy. In addition to diagnosis, the management of a newly diagnosed AC during pregnancy is also challenging, as data are limited to the ‘classical’ AC form in which RV is predominantly involved. In this AC phenotype, the majority of pregnancies have a positive outcome, without significant differences in incidence of sustained ventricular arrhythmias and in heart failure onset compared with a control group of nulliparous affected women.^9,10^ As our patient showed an AC with left dominant phenotype, we could speculate that the behavior during pregnancy could be similar to that of DCM patients. It is well known that pregnancy could be poorly tolerated in some women with DCM, with the potential for significant deterioration in LV function and that predictors of maternal mortality are NYHA class III/IV and ejection fraction less than 40%.^6^ None of these negative-predictive factors was present in our case.

As far as the genetic test result is concerned, *FLNC* gene mutations have been identified in patients with ‘classical’ right dominant AC, in left dominant AC forms and in a DCM phenotype characterized by extensive nonischemic LV fibrosis and occurrence of life-threatening ventricular arrhythmias (VAs) and sudden cardiac death.^11–14^ Currently, no data on pregnancy tolerance in patients carrying *FLNC* gene mutations are available. Thus, even if existing studies seem to indicate that pregnancy is well tolerated in AC and DCM patients with NYHA I class and a mildly reduced ejection fraction, strict cardiac monitoring and accurate management in close cooperation with obstetricians are mandatory.

Regarding medical therapy, beta-adrenergic blocking agents are generally well tolerated in pregnancy, even if they can be associated with increased rates of fetal growth restriction and hypoglycemia. Beta-1-selective drugs, such as metoprolol, are preferred as they are less likely to affect uterine contraction and peripheral vasodilation, and they have shown lower rates of fetal growth retardation. On the contrary, angiotensin-converting enzyme inhibitors and angiotensin receptor blockers are contraindicated.^6^

As far as ICD indication is concerned, current guidelines mainly refer to classical right or biventricular forms of AC, although data on arrhythmic stratification in patients with AC with left dominant phenotype are still lacking. Recent studies reported that the risk of sudden arrhythmic death in carriers of truncated mutations in the *FLNC* gene is not related to LV systolic function. Following the 2019 Heart Rhythm Society (HRS) consensus in this type of patient, an ICD implantation is recommended (class IIa) as primary prevention in those with a mild reduction in LV ejection fraction (<45%).^15,16^ In our case, the presence of a presyncopal episode and frequent NSVT induced us to implant an ICD. Subsequently, results of genetic analysis supported our decision. Nonetheless, the correct timing of ICD implantation in our patient could be debatable, as it is known that it can be implanted safely, especially in the first 8 weeks of gestation.^6^

This case emphasizes the role of pregnancy in revealing a previously concealed cardiomyopathy and raises the issue of diagnostic and therapeutic strategies in pregnant women affected by newly diagnosed cardiac disease. Moreover, this report underlies the need for close cooperation between cardiologists and gynecologists in the clinical management of this kind of patient. Finally, it confirmed the central role of family history, CMR and genetic study both in diagnosis and treatment planning in patients with arrhythmic cardiomyopathy.

## Conflicts of interest

There are no conflicts of interest.
